# Perceived risk for HIV acquisition and sexual HIV exposure among sexual and gender minorities: a systematic review

**DOI:** 10.1186/s12879-024-09456-0

**Published:** 2024-06-10

**Authors:** Hamid Vega-Ramirez, Centli Guillen-Diaz-Barriga, Paula M. Luz, Thiago S. Torres

**Affiliations:** 1 Division of Epidemiology and Psychosocial Research, National Institute of Psychiatry Ramon de la Fuente Muñiz, Mexico City, Mexico; 2 Division of Graduate Studies and Research, Faculty of Psychology, National Autonomous University of Mexico, Mexico City, Mexico; 3 Instituto Nacional de Infectologia Evandro Chagas, Fundação Oswaldo Cruz (INI- Fiocruz), Rio de Janeiro, Brazil

**Keywords:** Perceived risk for HIV acquisition, Sexual HIV exposure, Sexual and gender minorities, Transgender people, Men who have sex with men

## Abstract

We conducted a systematic review to explore the relationship between perceived risk for HIV acquisition and sexual HIV exposure among sexual and gender minorities. We included 39 studies divided into (i) correlations or associations, (ii) models using sexual HIV exposure as the outcome, and (iii) models using perceived risk for HIV acquisition as the outcome. The sample size range was from 55 to 16,667 participants, primarily cisgender men who have sex with men (73.3%) and White (51.3%). Sexual HIV exposure and perceived risk for HIV acquisition assessments and recall time frames across studies differed markedly. Most of studies (84.6%) found significant correlations, comparisons, or associations between different levels of perceived risk for HIV acquisition and high sexual HIV exposure. In addition, 51.3% of studies reported other variables associated with high sexual HIV exposure (i.e., misuse of substances or alcohol) or with high perceived risk for HIV acquisition (i.e., younger age). In conclusion, the association between perceived risk for HIV acquisition and sexual HIV exposure has shown to be consistent. However, the assessment for perceived risk for HIV acquisition should include more components of perception (i.e., an affective component), or for sexual HIV exposure should consider the different estimated sexual per-acts probability of acquiring HIV.

**Supplementary Information:**

The online version contains supplementary material available at 10.1186/s12879-024-09456-0.

## Background

According to the Joint United Nations Program on HIV/AIDS (UNAIDS), 38.4 million people were living with HIV worldwide in 2023, of which 39.0 million were adults (aged 18 years or older) [1]. Data suggest transgender women (TW) are 34 times more likely to acquire HIV than the general population, while gay, bisexual, and other men who have sex with men are up to 25 times more likely to acquire HIV [2]. The UNAIDS Global AIDS Strategy (2021–2026) seeks to reduce the inequalities in the HIV epidemic through a comprehensive package of prevention services addressed to populations most vulnerable to HIV acquisition [3].

Behavior is one axis for HIV prevention. Hence, health behavior theories have tried to explain how prevention and decision-making behaviors are related and which barriers and facilitators influence people’s conduct when they choose to adopt (or not) HIV prevention strategies [4, 5]. Examples of health behavior theories are the health belief model [6], the protective motivation theory [7], and the theory of planned behavior [8]. All these theories have a common denominator: direct or indirect risk perception is thought to influence behavior. These theories propose that increasing people’s risk perception could reduce their risk behavior, yielding subsequent benefits to physical and mental health [9].

In health sciences, particularly medicine and public health, risk perception is understood as an individual’s subjective assessment of the probability of an undesirable outcome [10], and it has a cognitive and an affective component [4, 11, 12]. The cognitive component refers to the perceived likelihood of harm (i.e., it is the subjective probability of experiencing an adverse outcome given one’s behavior) and the perceived susceptibility to injury (degree of presumed liability arising from one’s conduct) [4]. These two cognitive dimensions have been the most studied components regarding risk perception. The affective component, which encompasses both anticipatory and anticipated risk-related emotions felt during risk evaluation and when facing consequences of risky decisions in the future, frequently takes precedence over a statistical analysis of the risks and benefits when making decisions [13, 14]. For example, contemplating the potential adverse outcomes of a risky activity can evoke negative emotions and stress, reducing individuals’ willingness to engage in perceived risky behaviors. Therefore, understanding how the affective component influences risk perception is crucial for developing effective risk management strategies and promoting health behaviors [15].

It is well-known that the perceived risk for HIV acquisition and sexual HIV exposure^1^ (i.e., one’s sexual behavior) may not be aligned [12, 16–18]. Therefore, it is necessary to explore how perceived risk for HIV acquisition and sexual HIV exposure have been assessed to understand their relationship better. This systematic review sought to synthesize the literature on studies evaluating the relationship between perceived HIV risk and sexual HIV exposure among sexual and gender minorities.

## Methods

### Protocol and registration

This study was registered in the International Database of Systematic Reviews in Health and Social Care (PROSPERO 2021 CRD42021278247), and it is reported according to the Statement of Preferred Reporting Items for Systematic Reviews and Meta-Analyses (PRISMA) [19].

### Eligibility criteria

We included studies in English, Portuguese, or Spanish published from 1981 (when HIV was identified as a worldwide public health outbreak) to July 2023 with the following criteria: (i) participants were adults (≥ 18 years), (ii) sexual and gender minorities of any gender; (iii) cisgender men who have sex with men (cis-MSM) and do not identify as gay or bisexual, (iv) had unknown or negative HIV serostatus, (v) perceived risk for HIV acquisition and sexual HIV exposure were simultaneously assessed, (vi) reported correlation, comparison or association (unadjusted or adjusted) between perceived risk for HIV acquisition and sexual HIV exposure. We excluded reviews, meta-analyses, thesis, dissertations, monographs, conference papers and reports, qualitative studies, or studies that included injection drug users or reported grouped results with other populations different from sexual and gender minorities.

### Information sources

We performed a literature search on MEDLINE, IBECS, LILACS, CUMED, LIPECS, medRxiv, LIS (Localizador de Informação em Saúde), Coleciona SUS, BIGG-guias GRADE, PAHO-IRIS, COCHRANE, and SciELO.

### Search strategies

The search combined terms derived from five domains: (a) perceived risk for HIV acquisition, (b) sexual HIV exposure, (c) HIV, (d) sexual minority, and (e) gender minority. We used PubMed, Embase, and Lilacs to perform the research. Search keys are available in the Additional file 1. All studies were exported to Zotero software, and duplicates were excluded. The last date we performed the search was July 15th, 2023.

### Selection process

Two authors reviewed all abstracts independently, according to the eligibility criteria, and another author reviewed discrepancies to agree on the final list of full-text articles to be reviewed. Authors attempted to reach corresponding authors to request full manuscripts when unavailable. All authors reviewed all articles independently and discussed discrepancies until they agreed.

### Data collection process

We collected data using structured Excel spreadsheets. Before data collection, investigators discussed which variables should be collected, considering the main objective of this study.

### Data items

Data collected from the selected studies included author(s), year, country, recruitment strategy, study period, sample size, age, gender (self-reported gender identity, regardless of participants’ sex assigned at birth), race/ethnicity, sexual HIV exposure assessment (including recall time), perceived risk for HIV acquisition assessment (including recall time), statistical analyses, and significant findings resulting from comparisons and correlations, as well as unadjusted and adjusted associations estimated with regression models.

### Synthesis methods

We divided the selected studies into three groups depending on the analysis performed or the outcome used for multivariable models: (i) correlations or associations studies, (ii) logistic regression or Poisson robust error models using sexual HIV exposure as the outcome, and (iii) logistic regression models using perceived risk for HIV acquisition as the outcome. We also extracted data on other factors associated with perceived risk for HIV acquisition and sexual HIV exposure when available.

## Results

### Study characteristics

**Fig. 1:**
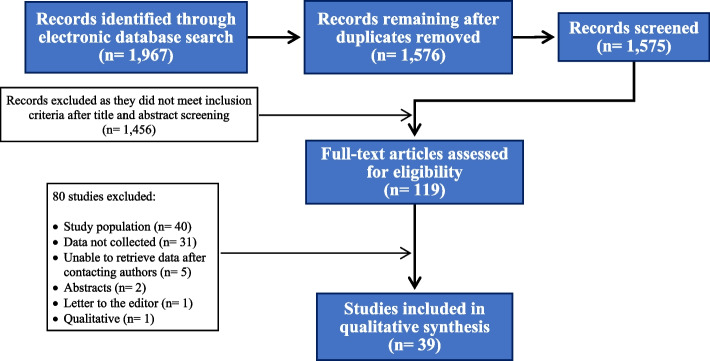
Flow diagram of study selection for the review of perceived risk for HIV acquisition and sexual HIV exposure among sexual and gender minorities

The flow diagram of the selection process is shown in Fig. 1. We identified 40 studies [20–59] carried out in 18 countries, but two studies were considered as one since they used the same sample but did complementary analysis [43, 56] (final sample = 39). Most studies were carried out in the United States of America and Canada (13; 33.3%), followed by Asia (11; 28.2%), Latin America (7; 17.9%), Europe (5; 12.8%), and Africa (2; 5.1%). One (2.6%) study was conducted on multiple continents [27].

All studies were written in English, but one written in Portuguese [26]. Six studies (15.4%) did not specify the study period; the remaining were conducted between 1991 and 2020. Twenty-four studies (61.5%) were carried out before September 2015, when the World Health Organization (WHO) recommended PrEP for all key populations [60]. Recruitment in LGBTQIA + venues and community-based organizations was more frequent (11, 28.2%), followed by online recruitment (10, 25.6%), HIV or STI health clinics (7; 17.9%), respondent-driven sampling (5; 12.8%), and advertisements on magazines (1; 2.6%). Five studies (12.8%) used two or more methods for recruitment. Only one study had a longitudinal design (from 1999 to 2018 – before and after WHO’s PrEP recommendation) [22]; all other studies were cross-sectional.

The sample size range from all studies was from 55 to 16,667. Sixteen studies (41%) reported mean age (from 19.7 to 39 years), 14 (35.9%) reported median age (from 20.4 to 44.5 years), seven (17.9%) defined age groups, and two (5.2%) did not provide information. Most studies included only cis-MSM (29; 73.3%), three only TW (7.7%) [28, 30, 31], five included cis-MSM and TW (12.8%), and two included cis-MSM, TW and non-binary people or other genders (5.2%) [57, 59]. Almost half of the studies (17; 43.6%) enrolled participants from different races or ethnicities, followed by those with participants from only one ethnicity: two (5.1%) with White [54, 55], two (5.1%) with Black [45, 50], two (5.1%) with European [22, 35], and one (2.6%) with Asian [39]. White people were the most prevalent group across the studies (20, 51.3%). Fifteen studies (38.5%) did not provide information about participants’ ethnicity.

### Sexual HIV exposure assessment

Sexual HIV exposure assessments across studies varied considerably. Only seven studies (17.9%) used a validated instrument or scale (HIV Incidence Risk Index for MSM [61, 62]), two (5.1%) used study-specific scales, one (2.6%) used a single question, and 29 (74.4%) considered a single or a combination of different sexual HIV exposures from a given list (i.e., condomless receptive or insertive anal sex, inconsistent condom use, sex with a person living with HIV, or previous HIV tests, among other options). Recall time frames across studies were also different: last 12 months (6, 15.4%), six months (20; 51.3%), three or fewer months (8, 20.5%), and other (4, 10.2%). Only one study did not specify the time frame for the recall (2.6%). See columns *Sexual HIV exposure* for detailed information in Tables 1, 2 and 3.

**Table 1:** Studies with comparison or correlation analysis between sexual HIV exposure and perceived risk for HIV acquisition (*n* = 10)

Author, year	Country	Recruitment	Study period	*N*	Age, years median (IQR), mean (SD) or strata (%)	Gender	Race/ ethnicity	Sexual HIV exposure		Perceived risk for HIV acquisition		Statical analysis	Comparison / Correlation
								Assessment	Recall time	Assessment	Recall time		
Bennett, 2020 [23]	USA	Online, multiple social media	2014	341	22 (IQR:20–23) Range: 18–24	Cis-MSM	15% Black 42% Hispanic 42% White	UAI casual or transactional partners; median # casual or transactional partners	Prior year	Single question: “How likely is it that you are infected with HIV, but might not know it?” Responses: not possible, not likely, and likely (combination of somewhat likely, likely and very likely).	Current	Chi-square test, correlation of perceived and risk	No correlation: UAI casual partners (*p* = 0.29); UAI with exchange partner (*p* = 0.33); median number of casual partners (*p* = 0.10); median number of transactional partners (*p* = 0.50).
Blumenthal, 2019 [24]	USA	HIV testing sites	2014–2016	171	32 (IQR:25–42)	Cis-MSM	60% White 29% Latino 8% Black	HIRI-MSM scale (7-items; scores: 0–47; 10 + points: high risk); CalcR score (16-items), Categories: low, moderate, high, very high	HIRI MSM prior 6 mo.; CalcR score: pior mo.	SPR: 3-items; score range: 0–13, categorized in: Low (0–3), Moderate (4–6), High (7–9), and Very High (10–13).	Next year	Cross-tabulation; Cohen’s Kappa Coefficient risk.	CalcR vs. SPR: 49% Concordant; 38% Underestimated risk; 13% overestimated risk (Kappa = 0.176) HIRI-MSM vs. SPR: 51% Concordant; 44% Underestimated risk; 5% overestimated risk (Kappa = 0.053)
De Santis, 2017 [30]	USA	HIV testing/ counseling and mental health centers, university-based gender reassignment surgery clinic	NS	50	38.4 (SD:14.8); Range: 20–78	TW	36% White 46% Latino 18% Black	Behavioral Risk Assessment Tool, 35-itens sexual behavior. Score:0–35.	Prior 3 mo.	Perceived Risk for HIV Infection Scale (4-item). Responses: 1 = “very unlikely” to 5 = “very likely.” Score: 4–20.	Current	Correlation coefficients between risk perception and behavior.	No statistically significant relationship between HIV risk perception and HIV risk behaviors (*r* = 0.157, *p* = 0.352),
Halkitis, 2004 [32]	USA	Peer-recruitment and advertisements at gay venues and publications	NS	97	39 (SD:10.7); Range: 20–70	Cis-MSM	14% Black 68% White 9.3% Latino 8.2% other	URAI, UIAI, UROI, UIOI	Prior 4 mo.	Extent to which they attributed their HIV- status: “Low probability of getting HIV” (1-item). Response ranged from 1 (“Has not contributed at all to keeping me negative”) to 4 = (“Has contributed a lot to keeping me negative”).	NS	ANOVA	Low probability of getting HIV and URAI with HIV-/unknown casual partners [F(1, 83) = 4.35, *p* = 0.04). Means of URAI (agreement vs. not): 7.79 (SD = 15.80) vs. 2.45 (SD = 7.35). No significance between low probability of getting HIV with URAI, UIAI, UROI, UIOI with casual sexual PLWH.
Herder, 2020 [35]	Sweden	Attending 6 HIV testing venues	2018	658	32 (IQR:27–41)	98.9% Cis-MSM 1.1% TW	63.9% Sweden 18.3% other European country 17.8% out of Europe	# URAI; # male sex-partner.	Prior 12 mo.	“What do you believe your current risk of getting HIV is?”, No risk, Low risk, Moderate to high risk.	Current	Chi Square comparing risk and perceived risk	High # URAI associated with moderate/high perceived risk (*p* = 0.014); High # male sex-partners (*p* = 0.006).
Jaspal, 2019 [36]	UK	Online (Grindr)	NA	191	33.6 (SD:11.2)	Cis-MSM	74% White British 9% South Asian 8% Other white	Engagement in sex-risk behavior using 3-items: frequency of cursing, use app and sauna to find sex-partners (6-Likert Scale: 1-never to 6- every day). Scores: 3–18	Prior mo.	HIV perceived risk scale (Napper 2012); 8-item	NA	Spearman’s rho correlations	Sex-risk behavior correlated with HIV perceived risk (*r* = 0.44; *p* < 0.001). Perceived risk in the pathway of sex-risk behavior (beta = 0.31; *p* < 0.005) in a SEM pathway model to evaluate HIV knowledge and PrEP acceptability.
Kesler, 2016 [37]	Canada	HIV prevention/ treatment clinic	2010–2012	150	44.5 (IQR:37–50)	Cis-MSM	82.9% White 17.1% Other	HIRI-MSM	Prior 6 mo.	‘What do you think the chances are that you will ever get HIV/AIDS?”; dichotomized in “High” (somewhat likely, very likely) vs. “Low” (impossible, not likely).	NS	Chi-square test	Risk and HIV perceived risk were associated (*p* = 0.020); however, 56/77 (72.7%) of those in high risk perceived themselves in low risk.
Pico- Espinosa, 2023 [48]	Canada	Dating apps, social media, and sexual health clinics	2019–2020	488	35 (IQR: 27–48)	Cis-MSM	59.9% White 10.8% Asian 5.7% Latin 4.1% Black 2.2% Indigenous 17.2% Other	HIRI-MSM	Prior 6 mo.	“How would you rate your risk for HIV infection in the next year?”, with response options “low”, “high” or “unsure”.	Next year	Chi square, Fisher’s exact or Wilcoxon rank-sum tests, comparing concordant (C) vs. discordant (D) groups according to their perceived and actual HIV exposure	HIRI-MSM median, (IQR): C = 6 (3–8), D = 18 (15–21) (*p* < 0.001); number sexual partners, (IQR): C = 2 (1–3), D = 3 (1–5) (*p* < 0.001); number sexual partners PLWH, (IQR): C = 0 (0–0), D = 0 (0–1) (*p* < 0.001); ever syphilis diagnosis: C = 15 (12%), D = 26 (26%) (*p* = 0.012); ever gonorrhea diagnosis: C = 37 (27%), D = 54 (44%) (*p* = 0.003); last year gonorrhea diagnosis: C = 13 (10%), D = 21 (19%) (*p* = 0.035); ever rectal gonorrhea diagnosis: C = 5 (14%), D = 22 (48%) (*p* = 0.002); previous 6 mo. methamphetamine use: C = 1 (1%), D = 13 (9%) (*p* < 0.001); previous 6 mo. poppers use: 29 (17%), D = 50 (34%) (*p* < 0.001)
Steiner, 1994 [55]	USA	Gay bar	1991	434	32 (SD: NS)	Cis-MSM	83.9% White	UAI	Prior 2 mo.	Question not described. Responses: 1-not at all, slight, some, and 4-a lot.	Prior 2 mo.	ANOVA using HIV perceived risk as outcome	Engage in UAI: 2.2 (SD:0.9) Not engage: 1.5 (SD:0.7); *p* < 0.001
Torres, 2021 [57]	Brazil	Hornet, Grindr, Facebook, Instagram	2020	4107	32 (IQR:26–40)	97.4% Cis-MSM 2.6% trans or non-binary	57.4% White 11.5% Black 31.1% Pardo or Indigenous	HIRI-MSM UAI # male partners	Prior 6 mo.	8-item Perceived risk of HIV Scale (Napper 2012)	NA	t-test	HIRI-MSM: high: 26.7 (SD:4.8); low: 24.4 (SD:4.8); *p* < 0.001 UAI: yes: 27.0 (SD:4.7); no: 25.0 (SD:4.9), *p* < 0.001; > 5 partners: 27.0 (SD:4.7); ≤5 partners: 24.8 (SD:4.8), *p* < 0.001

*AI* Anal intercourse, *CalcR* Calculated HIV risk score, *CCD* Consistent condom use, *ICD* Inconsistent condom use, *IQR *Interquartile range, *HIRI-MSM* HIV Incidence risk index for MSM, *cis-MSM * Cisgender men who have sex with men, *NA* Not applicable, *NS * Not specified, *PLWH * People living with HIV, *SD* Standard deviation, *STI*  Sexually transmitted infections, *TW*  Transgender women, *UAI*  Unprotected anal intercourse, *UIAI*  Unprotected insertive anal intercourse, *UIOI*  Unprotected insertive oral intercourse, *URAI*  Unprotected receptive anal intercourse, *UROI*  Unprotected receptive oral intercourse

**Table 2:** Studies with Simple or Multiple Regression Models to Assess Factors Associated to Sexual HIV Exposure (*n* = 19)

Author, year	Country	Recruitment	Study period	*N*	Age, years median (IQR), mean (SD) or strata (%)	Gender	Race/ ethnicity	Sexual HIV exposure		Perceived risk for HIV acquisition		Unadjusted Association OR (95% CI)	Adjusted Association aOR (95% CI)
								Assessment	Recall time	Assessment	Recall time		
Aho, 2014 [20]	Cote D’Ivoire	RDS	2011–2012	601	23 (IQR:18–51)	Cis-MSM	NS	UAI: not having consistently used a condom for receptive or insertive anal sex with any regular or casual male partner	Prior year	Self-perceived HIV risk (question not described): no, low risk, high risk, I don’t know, I am HIV positive.	NS	Low (ref. no risk): 3.02 (1.65–5.55); high risk: 8.50 (3.72–19.39); don’t know: 0.58 (0.06–5.96)	Low (ref. no risk): 2.14 (1.11–4.11); high risk: 6.00 (2.31–15.63)
Brignol, 2011 [26]	Brazil	Gay magazines, flyers in gay venues; gay NGO websites	2003–2006	533	30.5 (SD:9.5)	Cis-MSM	71.5% White 28.5% Black or Pardo	UAI with PLWH or unknown HIV status partner	Prior year	“Feel at risk of acquiring HIV/AIDS”; response categories: low, medium, and high (dichotomized as low and medium/high)	NS	Medium/high HIV perceived risk (ref. low): 2.69 (1.83–3.94)	Medium/high HIV perceived risk (ref. low): 2.33 (1.53–3.57)
Chen, 2023 [28]	China	Non-governmental organization, snowball sampling	Nov- 2018 to Jan-2019	247	< 30 (41.3%) 30–45 (49.4%) > 45 (9.3%) Range: 18–61	TW	NS	UAI, multiple sexual partners (regular and irregular), commercial sexual partners	Prior 6 mo.	Self-composed 5-point Likert scale (*favorable* to *unfavorable*) with 4 items. Higher score equals low perceived risk for HIV acquisition	NS	For UAI = Higher perceived risk for HIV acquisition: 0.80 (0.72–0.89)	NS
Choi, 2004 [29]	China	Informal social networks and peer-recruiters at MSM venues	2001–2002	482	Mean: 27 (SD: NS) range:18–69	Cis-MSM	NS	UIAI, URAI	Prior 6 mo.	“What do you think about your own risk for developing AIDS? Would you say your risk is none, low, somewhat high, or very high?”	NS	For UIAI = High (ref. low): 2.03 (1.22–3.36) For URAI = High (ref. low): 1.43 (0.86–2.37)	For UIAI = High (ref. low): 1.61 (0.94–2.76) For URAI = Not included in final model
Hentges, 2023 [34]	Brazil	RDS	2016	2722	26.1 (SD:0.40)	Cis-MSM	34.7% White 65.3% Non-White	ICU with answers in 4-point Likert scale (never, rarely, sometimes, always). It was created using frequency of condom use in all receptive and insertive anal intercourse, and in the last sexual encounter	Prior 6 mo.	Self-reported current risk of HIV infection with responses: none/low or moderate/high	NS	Medium/high self-reported current risk of HIV infection: 1.52 (1.11–2.10)	Medium/high self-reported current risk of HIV infection: 1.51 (1.07–2.14)
Khumsaen, 2017 [38]	Thailand	Online (Facebook)	2015	469	19.7 (SD:1.13) range:18–21	Cis-MSM	NS	UAI; never HIV test; partner with HIV unknown status; drunk sex	Prior 6-mo. (except for HIV test)	1st domain of the AIDS Health Belief Scale (perceived susceptibility). Scores: 4–20; 4-item; responses: 1-strongly disagree to 5-strongly agree.	NA	For partner with unknown HIV status = High: 0.89 (0.81–0.96)	NA
Koh, 2014 [39]	Malasya	Community-based	Jan/ Dec-2008	423	Mean: 29.2 (range: 18–61)	Cis-MSM	60.0% Chinese 27.4% Malays 4.5% Indian 8.4% Other	> 10 sex-partners, > regular sex-partner, alcohol use before sex, last UAI, ICU.	Prior 6 mo.	Question not described; responses were: “low risk” (< 25% chance), “medium risk” (25–75%), “high risk” (> 75%), and “unsure risk.”	Next year	For > 10 sex-partners = High risk: 42.0 (8.83-258.47), For > regular sex-partner = High risk: 10.67 (3.13–36.36), For alcohol use before sex = High risk: 10.67 (3.13–36.36), For last UI = High risk: 10.67 (3.12–6.42), For ICU = High risk: 12.18 (3.80-39.03)	NA
Lau, 2013 [40]	China	MSM venues	2007–2008	195	< 30 (31.8%) 31–40 (41.0%) > 40 (27.2%)	Cis-MSM	NS	UAI with any man; UAI with casual or commercial partner	Prior 6 mo.	Question not described; perceived chance in contracting HIV stratified definitely no chance and some chance	Next year	For UAI with any man = some chance: 5.10 (*p* < 0 0.001); For UAI with casual or commercial = moderate perceived risk: 4.89 (*p* < 0.001)	For UAI with any man: some chance = 10.37 (3.26–33.04); For UAI with casual or commercial = moderate perceived risk 3.87 (1.58–9.53)
Lau, 2014 [41]	China	Gay venues and online (Hong Kong only)	NS	535	18–24 (35.3%) 25–34 (53.0%) 35–52 (11.7%)	Cis-MSM	NS	UAI	Prior 12 mo.	Question not described; perceived chance of contracting HIV in the future (extremely low/low vs. moderate/high/ extremely high)	NS	High: 4.63 (1.92–11.18) (only for Shezhen, but not for Hong Kong participants)	High: 3.91 (1.57–9.70) (only for Shezhen participants)
Li, 2017 [42]	China	RDS	2013–2014	459	30 (IQR:25–39)	Cis-MSM	NS	UAI	Prior 6 mo.	Question not described; self-perceived likelihood of HIV infection	NS	Perceived risk was not associated	NA
Maksut, 2016 [45]	USA	Gay venues; online classifieds and social media (e.g. Facebook, Black Gay Chat, Jack’d)	2012–2014	450	YMSM: 24.0 (SD:3.0) OMSM: 43.9 (SD:8.4)	Cis-MSM	Black	UAI	Prior 3 mo.	5 -items: e.g. “How risky is anal sex without condom as the bottom partner with a man you just met who tells you his HIV status is negative?”. Responses: 0-no/low risk to 10-very high risk. Score: 0–50.	NS	For YMSM = RR: 0.93 (0.85–1.02); For OMSM = RR: 0.76 (0.72–0.83)	For YMSM: perceived risk not included in final model For OMSM = aRR: 0.85 (0.78, 0.93)
Morell- Mengual, 2021 [46]	Spain	Online; advertisements on NGO social media and website	NS	349 MSM 56 MSMW	28.9 (SD:9.35) range: 18–60	Cis-MSM	NS	Item #35 AIDS Prevention Questionnaire “How often have you used a condom during anal sex with casual partners?” Responses: do not have this practice, never sometimes, quite often or always.	NA	Perceived vulnerability to HIV (AIDS Prevention Questionnaire)	NA	NS	1.02 (1.01–1.03)
Pham, 2015 [47]	Vietnam	Community-based	2009	381	20.4 (IQR:18-25.1)	Cis-MSM	NS	Multiple UAI partners	Prior mo.	NS	NS	Low (ref. no risk/ I don’t know): PR:1.16 (0.84–1.59); high: PR: 2.03 (1.42–2.91)	Low (ref. no risk/ I don’t know): aPR:1.04 (0.77–1.41); high risk: aPR:1.81 (1.32–2.49)
Raymon, 2009 [50]	Uganda	RDS	2004	215	NA	Cis-MSM	Black	URAI	Prior 6 mo.	Self-perception of risk for HIV infection (None, low, somewhat high, high)	NS	NS	Perceived risk was not associated
Rocha, 2020 [51]	Brazil	RDS	2016	4129	< 25 (60.6%) 25+ (39.4%)	Cis-MSM	34.8% White 65.2% Non-white	URAI	Prior 6 mo.	Self-reported current risk of HIV infection (None/Low or Moderate/High)	NS	For < 25 years = moderate/high (ref. none/low): 1.83 (1.18–2.85); For 25 + years = moderate/high (ref. none/low): 2.29 (1.26–4.16)	For < 25 years: moderate/high (ref. none/low): 1.75 (1.09–2.82); For 25 + years: not included in final model
Sharma, 2018 [53]	USA	Online (Facebook)	2015	800	NA	Cis-MSM	80.3% non-Hispanic white 8.1% non-Hispanic non-white A11.6% Hispanic	UAI ≥ 2 men	Prior 3 mo.	Overall concern about contracting HIV; Score: 0–10.	NS	NS	Concern about contracting HIV was not significant
Stack, 2016 [54]	Canada and USA	Online; e-mail to members of a popular social networking site for MSM	2011	3217	40 (IQR:28–49)	Cis-MSM	84% White	UAI at least once and frequent UAI (> 1x/week)	Prior 3 mo.	“Based on your sexual experiences in the past 3 months with male sex partners, if you were to rate your risk of getting HIV on a scale of 1–10, with 1 being not risky at all to 10 being extremely risky, how would you rate yourself?”	Prior 3 mo.	For UAI at least once = High: 1.28 (1.24–1.30); For frequent UAI= High: 1.12 (1.07–1.18)	For UAI at least once = High: 1.26 (1.22–1.31); For frequent UAI = High: 1.17 (1.11–1.23)
Vargas, 2018 [58]	Peru	STI clinics	2013–2014	310	18–25 (32.9%) 26–35 (35.2%) ≥36 (31.9%)	78% Cis-MSM 22% TW	NS	Frequent HIV testing	At least every 6 mo.	Question not described. Responses: high, moderate, low or no risk.	NS	No/low perceived risk (ref. moderate) = PR: 1.87 (1.37–2.55) High = PR: 1.31 (0.89–1.92)	No/low = aPR: 1.53 (1.13–2.08) High = aPR: 1.02 (0.71–1.46)
Yi, 2015 [59]	Cambodia	Venues and hotspots identified by community health workers	2014	367	23.9 (SD:5.2)	56.4% Cis-MSM 21.5% TW 22.1% Other	NS	ICU	Prior 3 mo.	Question not described; self-perception of HIV risk compared to the general population (higher, same, lower)	NS	NS	Higher (ref. same): 2.37 (1.35–4.17) Lower: 0.75 (0.39–1.47)

*AI* Anal intercourse, *CCD* Consistent condom use *ICU *Inconsistent condom use, *IQR *Interquartile range, *cis-MSM *Cisgender men who have sex with men, *MSMW *Men who have sex with men and women, *NA *Not applicable, *NS *Not specified, *OMSM *Old men who have sex with men, *PLWH *People living with HIV, *RDS *Respondent driven sampling, *SD *Standard deviation, *STI *Sexually transmitted infections, *TW* Transgender women, *UAI *Unprotected anal intercourse, *UIAI *Unprotected insertive anal intercourse, *UIOI *Unprotected insertive oral intercourse, *URAI *Unprotected receptive anal intercourse, *UROI U*nprotected receptive oral intercourse, *YMSM *Young men who have sex with men

**Table 3:** Studies with Simple or Multiple Regression Models to Assess Factors Associated to Perceived Risk for HIV Acquisition (*n* = 10)

Author, year	Country	Recruitment	Study period	*N*	Age, years median (IQR), mean (SD) or strata (%)	Gender	Race/ ethnicity	Sexual HIV exposure		Perceived risk for HIV acquisition		Unadjusted Association OR (95% CI)	Adjusted Association aOR (95% CI)
								Assessment	Recall time	Assessment	Recall time		
Alexovitz, 2018 [21]	USA	Online; multiple social media websites	2015–2017	2275	22 (IQR:21–23) Range: 18–24	Cis-MSM	44.7% White 36% Hispanic 19.3% Black	UAI with man; UAI main male partner; UAI casual male partner	Ever	Possibility of acquiring HIV (question not described): “not possible at all”, “not likely”, “somewhat likely”, “likely”, or “very likely”, then dichotomized in “not possible at all” vs. “other”	NS	UAI with man: 2.10 (1.61–2.73); UAI main male partner: 1.31 (1.06–1.61); UAI casual partner: 1.82 (1.48–2.24)	NA
Basten, 2021 [22]	Netherlands	Brochures at STI clinic, advertisements in the gay scene, and chain referral sampling	1999–2018 Longitudinal cohort study; 40 waves	1323	31.3 (SD:9.9) at cohort inclusion	Cis-MSM	80.5% Dutch 15.9% 1st generation immigrants 3.6% 2nd generation immigrants	# casual partners IAI; # casual partners RAI; UAI casual partner; no UAI steady partner; UAI steady partner; URAI PLWH	Prior 6 mo.	Participants were asked to rate the likelihood to acquire HIV (7-point Likert scale: 1: “impossible” to 7 “very likely” (categories 5–7 were combined).	Next 6 mo.	1999–2003= # casual partners IAI: 2.44 (2.11–2.82); # casual partners RAI: 2.56 (2.21–2.97); UAI casual partner: 6.94 (5.43–8.88); UAI steady partner: 0.75 (0.59–0.95) 2004–2008: # casual partners IAI: 2.42 (2.14–2.73); # casual partners RAI: 2.42 (2.13–2.74); UAI casual partner: 6.25 (5.02–7.77); UAI steady partner: 0.65 (0.52–0.80); URAI PLWH: 8.83 (4.23–18.46) 2008–2011= # casual partners IAI: 1.97 (1.68–2.32); # casual partners RAI 2.20 (1.86–2.61); UAI casual partner: 4.12 (3.07–5.52); UAI steady partner: 0.64 (0.46, 0.89); URAI PLWH: 6.57 (2.87–15.04) 2011–2016= # casual partners IAI: 1.90 (1.75–2.07); # casual partners RAI: 1.92 (1.75–2.10); UAI casual partner: 4.59 (3.91–5.39); UAI steady partner: 0.43 (0.36–0.52); URAI PLWH: 2.66 (1.79–3.95) 2017–2018= # casual partners IAI: 1.77 (1.54–2.03); # casual partners RAI: 1.69 (1.46–1.94); UAI casual partner: 4.08 (3.08–5.40); UAI steady partner: 0.41 (0.30–0.58); URAI PLWH: 1.16 (0.72–1.89)	1999–2003= # casual partners IAI: 1.48 (1.24–1.76); # casual partners RAI: 1.78 (1.49–2.12); UAI casual partner: 4.66 (3.54–6.14); UAI steady partner: 1.54 (1.18-2.00) 2004–2008= # casual partners IAI: 1.60 (1.39–1.84); # casual partners RAI: 1.60 (1.38–1.85); UAI casual partner: 3.96 (3.11–5.03); UAI steady partner: 1.17 (0.92–1.48); URAI PLWH: 7.17 (3.26–15.76) 2008–2011= # casual partners IAI: 1.36 (1.14–1.64); # casual partners RAI 1.61 (1.33–1.94); UAI casual partner: 2.75 (1.99–3.81); UAI steady partner: 1.08 (0.76–1.54); URAI PLWH: 3.81 (1.58–9.16) 2011–2016= # casual partners IAI: 1.31 (1.19-0.45); # casual partners RAI: 1.33 (1.20-0.47); UAI casual partner: 2.98 (2.50–3.56); UAI steady partner: 0.72 (0.59–0.87); URAI PLWH: 1.89 (1.27–2.82) 2017–2018= # casual partners IAI: 1.31 (1.11–1.54); # casual partners RAI: 1.24 (1.05–1.47); UAI casual partner: 2.79 (2.01–3.88); UAI steady partner: 0.63 (0.45–0.88); URAI PLWH: 0.63 (0.38–1.05)
Bosga, 1995 [25]	Netherlands	Gay magazine	1991–1992	164	39 (SD:9.8), range: 21–68	Cis-MSM	NS	UAI with steady and any partner; ever had sex with PLWH	Prior 6 mo.	Single question: “How high do you estimate the risk to be that you have actually become infected with the AIDS virus/HIV in the last six months?”; 6-Likert scale (1 = high, 6 = no risk), then two categories created: underestimators (UAI and low/ very low perceived HIV risk) and acknowledgers (UAI and other responses)	Prior 6 mo.	NA	Ever had sex with a person living with HIV: *β* = 0.24, *r* = 0.22, *p* < 0.01; more defensive denial: *β* = 0.23, *r* = 0.22, *p* < 0.01; not being religiously active: *β*= -0.18, *r*= -0.19, *p* < 0.05
Chard, 2017 [27]	Australia Brazil Canada S. Africa Thailand UK USA	Online, Facebook	NS	274 294 274 386 146 280 254	30.4 (SD:11.5) 25.8 (SD:7.7) 33.9 (SD:12.6) 33.2 (SD:10.3) 32.3 (SD:7.8) 30.6 (SD:11.2) 30.9 (SD:13.4)	Cis-MSM	Minority: 38.7% Minority: 35.7% Minority: 20.1% Minority: 85.5% Minority: 5.5% Minority: 5.0% Minority: 18.5%	UAI	Prior year	Single question: “How would you rate your risk of contracting HIV based on your current behavior?” 1 (no risk) to 10 (very high risk).	Current	NA	0.87 (0.40–1.88) 0.92 (0.49–1.75) 0.93 (0.44–1.96) 1.76 (1.02–3.05) 1.65 (0.74–3.70) 1.50 (0.76–2.94) 0.63 (0.28–1.45)
Guillen-Diaz, 2023 [31]	Mexico	Online and public health clinic	2018	191	30 (IQR:24–37)	TW	NS	Number of sexual partners, URAI, UIAI, sex with PLWH, sex with partners with unknown HIV status, sex under the influence of alcohol, chemsex and, transactional sex.	Prior 6 mo.	One question: Considering your sexual practices, in your opinion, what would be your risk of getting HIV in the next 12 months? 3-point Likert scale (none, low, high)	Next year	> 5 sexual partners: 12.0 (4.4–32.4); UIAI: 6.3 (2.2-17-7); sex with PLWH: 15.2 (3.9–59.5); sex with partners with unknown HIV status: 6.5 (2.5–16.9); transactional sex: 9.9 (3.8–26.1); sex under the influence of alcohol: 3.7 (1.5–9.2)	> 5 sexual partners: 6.00 (1.1–31.2); URAI: 0.15 (0.3–0.8); sex with partners with unknown HIV status: 8.9 (2.0-38.5); transactional sex: 6.4 (1.3–31.2)
Hall, 2018 [33]	USA	Outreach recruitment at popular places for MSM.	2010–2012	324	18–28 (38.9%) 29–40 (36.4%) 41–48 (15.1%) 49+ (9.6%)	Cis-MSM	47.2% White 26.5% Hispanic/ Latino 10.8% Asian/ Pacific islanders 9.6% Black 5.9% Other	HIRI-MSM (7-item)	Prior 3 mo.	2-items: “Given my current sexual behavior, I can get infected with HIV”; “I am worried about becoming infected with HIV.” Response options: strongly agree to strongly disagree, then dichotomized into: agree or disagree.	NS	147 (45.4%) underestimated their HIV risk (low HIV perceived risk and high risk) 88 (27.2%) agreement between perceived and risk 89 (27.4%) overestimated the risk	Race: 0.87 (0.70–1.07); recently exchanged sex for drugs, money, or other goods: 2.56 (1.01–6.45); had a recent STI diagnosis: 2.34 (1.32–4.12); experienced more social isolation: 1.62 (1.01–2.60); substance dependent: 1.54 (0.97–2.46); experiencing more racial discrimination: 0.65 (0.39–1.09)
Luz, 2021 and Torres, 2019 [43], [56]	Brazil	Online (Hornet, Grindr, Facebook)	2016–2018	16,667	29 (IQR:24–36) 18–24 (26.6%) 25+ (73.4%)	Cis-MSM	58.1% White/ Asian 30.4% Non-White	URAI, previous STI, HIRI-MSM	Prior 6 mo.	“In your opinion, what is your risk of getting HIV in the next year?”; “No risk”, “Low risk”, “High risk/50%”, “Certain/100%” and “I don’t know / prefer not to answer”	Next year	High HIRI-MSM: 3.47 (3.19–3.78); URAI: 2.71 (2.52–2.91); > 5 male partners: 2.87 (2.67–3.09)	URAI: 2.54 (2.35–2.74)
MacKellar, 2007 [44]	USA	MSM venues	1998–2000	2788	23–25 (49.2%); 26–29 (50.8%)	Cis-MSM	49.6% White 24.0% Hispanic 18.6% Black 5.9% Asian 19.0% Mixed	UAI UAI with unknown HIV status partner	UAI (ever) UAI with unknown HIV status partner (prior 6 mo.)	“Using this card, choose a number that best describes how likely it is that you will become HIV positive in your lifetime” 5-point Likert scale (1-very unlikely to 5-very likely).	Ever	NA	For moderate/high= UAI with unknown HIV status partner: 2.25 (1.84–2.76) UAI: not included in final model
Plotzker, 2017 [49]	Thailand	Health facilities	2015–2016	297	25 (IQR:21.9–30.2)	54% TW 48% Cis-MSM	NS	ICU	Prior 6 mo.	Question not described. Answers: No risk, minimal, moderate or high.	NS	ICU: None: 9.2% Minimal: 36.0% Moderate: 33.6% High: 20.7% *p* = 0.015 ICU 1.76 (1.05–2.94);	NA
Seekaew, 2019 [52]	Thailand	Health facilities	2015–2016	1288	MSM risk discordant: 23.3 (20.5–28.5); risk concordant: 24.6 (20.8–29.3) TGW risk discordant: 23.1 (20.6–26.7); risk concordant: 24.1 (21.1–28.1)	882 MSM 406 TW	NS	At least one: tested HIV+, UAI, STI, amphetamine-type stimulants use, IDU, shared needles.	Prior 6 mo.	Participants were asked to rate their own HIV risk as: “No”, “Mild”, “Moderate” or “High”. Only low risk (no/mild) were included in this analysis.	NS	MSM: Being from Bangkok: 2.0 (1.2–3.3), or Chiang Mai: 3.2 (1.8–5.4); living with a male partner: 2.0 (1.2–3.1); ≤ bachelor’s degree: 1.5 (1.1–2.2); no previous HIV test: 1.5 (1.1–2.2); positive attitude about people living with HIV: 1.8 (1.0-3.1) TW: living with a male partner: 3.8 (1.5–9.9); <17 years at sexual debut: 2.2 (1.3–3.8); no male circumcision: 2.9 (1.2–5.6); <8 score of knowledge about HIV protection: 2.6 (1.5–4.4)	MSM: Being from Bangkok: 2.0 (1.2–3.4), or Chiang Mai: 2.8 (1.6–4.9); living with a male partner: 2.0 (1.2–3.2); no previous HIV test: 1.5 (1.0-2.1); positive attitude about people living with HIV: 1.9 (1.1–3.4) TW: living with a male partner: 5.6 (1.9–16.4); <17 years at sexual debut: 2.7 (1.5–4.9); sex worker: 0.5 (0.2–0.9); <8 score of knowledge about HIV protection: 2.9 (1.6–5.1)

*AI *Anal intercourse, *CCD *Consistent condom use, *IAI *Insertive anal intercourse, *ICD *Inconsistent condom use, *IDU *Intravenous drug user, *IQR *Interquartile range, *HIRI-MSM *HIV incidence risk index for MSM, *cis-MSM *Cisgender men who have sex with men, *NA N*ot applicable, *NS *Not specified, *PLWH *People living with HIV, *RAI *Receptive anal intercourse, *RDS *Respondent driven sampling, *STI *Sexually transmitted infections, *TW* Transgender women, *UAI *Unprotected anal intercourse, *UIAI *Unprotected insertive anal intercourse, *UIOI *Unprotected insertive oral intercourse, *URAI *Unprotected receptive anal intercourse, *UROI *Unprotected receptive oral intercourse

### Perceived risk for HIV acquisition assessment

Twenty studies (51.3%) used a single question with Likert-scale responses, and six (15.4%) used validated instruments or scales (Perceived Risk for HIV Infection Scale [63], HIV Perceived Scale [64], AIDS Health Belief Scale [65], AIDS Prevention Questionnaire [66]), and four (10.2%) study-specific scales. Eight studies (20.5%) did not specify the question but used Likert-scale responses, and one (2.6%) did not provide any information. Most studies (20; 51.3%) did not specify a time frame for perceived risk, but six studies (15.4%) considered the following year, four (10.2%) used the scales’ time frame (i.e., current time), four current perceptions (10.2%), and four studies used other time frames (10.2%). Only one study (2.6%) considered the perception of HIV acquisition across the lifespan [44].

### Association between sexual HIV exposure and perceived risk for HIV acquisition

Studies performed different analyses to evaluate and quantify the association between sexual HIV exposure and perceived risk for HIV acquisition. Ten studies (25.6%) performed simple comparison (*n* = 7/10) or correlation analysis (*n* = 3/10) (Table 1), and 29 studies (74.4%) performed a multivariate analysis; of these, 19 used sexual HIV exposure (19/29, 65.5%) (Table 2), and ten used perceived risks for HIV acquisition (10/29, 34.5%) (Table 3), as the outcome.

Six out of seven comparison studies were significant between high sexual HIV exposure and high perceived risk for HIV acquisition using Chi-squared test [35, 37, 48], ANOVA [32, 55], and *t-test* [57] (Table 1). Two [24, 36] out of three correlation studies found a significant positive correlation between high perceived risk for HIV acquisition and high sexual HIV exposure.

Table 2 depicted 19 studies [20, 26, 28, 29, 34, 38–42, 45–47, 50, 51, 53, 54, 58, 59] that used sexual HIV exposure as the outcome using univariable [28, 38, 39, 42], or multivariable logistic regression models, including perceived risk for HIV acquisition as a predictor. Three studies [28, 38, 39] with univariable regression analysis found that a high perceived risk for HIV acquisition was associated with lower odds of any sexual HIV exposure, and one did not find any association [42]. Conversely, twelve studies with multivariable logistic models found that those with a high perceived risk for HIV acquisition (reference: none or low) had higher odds of any sexual HIV exposure. Only one study using the same methodology found an inverse association, that is, lower relative risk for sexual HIV exposure (condomless anal sex) (aRR = 0.85 [0.78–0.93]), but only for MSM over 30 years old [45]. One study found the highest odds for this outcome (sexual HIV exposure) among those reporting some (aOR = 10.37 [3.26–33.04]) [40] and high perceived risk for HIV acquisition (aOR = 6.00 [2.31–15.63]) [20]. Two studies did not find an association between perceived risk for HIV acquisition and sexual HIV exposure [50, 53].

Ten studies used perceived risk for HIV acquisition as the outcome (Table 3). Two studies used simple regression models [21, 49], and seven studies used multivariable regression models and found that individuals reporting any sexual HIV exposure had higher odds of high perceived risk for HIV acquisition. Studies using simple regression models found that unprotected anal intercourse (OR = 2.10 [1.61–2.75]) [21] or inconsistent condom use (OR = 1.76 [1.05–2.94]) [49] increased the odds of high perceived risk for HIV acquisition. Studies using multivariable regression analysis also found that any sexual HIV exposure increased the odds of high perceived risk for HIV acquisition, such as having sex partners with unknown HIV status (aOR = 8.9 [2.0-38.5]) [31] for TW and having unprotected receptive anal intercourse with a person living with HIV (aOR = 7.17 [3.26–15.76]) [22] for cis-MSM. Bosga et al. [25] found that having sex with a person living with HIV (*β* = 0.24, *r* = 0.22, *p* < 0.01), high denial of risk for HIV acquisition (*β* = 0.23, *r* = 0.22, *p* < 0.01), and not being religiously active (*β*= -0.18, *r*= -0.19, *p* < 0.05) were associated with perceived risk for HIV acquisition.

Some multivariable studies (*n* = 21) reported other variables associated with sexual HIV exposure [20, 26, 28, 29, 34, 40, 41, 45–47, 50, 51, 54, 58, 59] or perceived risk for HIV acquisition [27, 31, 33, 43, 44, 56] (Table 4). Relevant variables that increased odds for sexual HIV exposure were: younger age (except for one study which found an association with age 25+) [59], low education level, high income, alcohol or substance misuse, never tested for HIV, increased number of sexual partners, transactional sex, previous STIs diagnosis, and history of sexual violence. Variables associated with high perceived risk for HIV acquisition were: younger age, low income or education (except for one study which found an association with high education level) [27], non-White race or ethnicity, alcohol or substance misuse, increased number of sexual partners, transactional sex, previous STIs diagnosis, PrEP eligibility or awareness, and post-exposure prophylaxis (PEP) awareness.

**Table 4:** Factors associated in multivariable models for sexual HIV exposure or perceived risk for HIV acquisition (*n* = 21)

Author, year	Factors associated with sexual HIV exposure (different from perceived risk for HIV acquisition)
Aho, 2014 [20]	History of forced sex (ref. no): 2.64 (1.23, 5.65) Alcohol use prior 30-d (ref. never): once a week: 2.05 (1.14, 3.69); more than once a week: 2.48 (1.13–5.44) One regular male anal sexual partner prior 12-mo (ref no): 1.93; (1.01, 3.66) Number of casual male anal sex partners in past 12mo (ref none): one: 2.04 (1.03, 4.01); two: 1.83 (0.81–4.13); 3+: 2.61 (1.22, 5.60) Transactional sex with men prior 12-mo: 6.15 (1.92, 19.74)
Brignol, 2011 [26]	Number of partners prior year: 1.71 (1.10–2.68); receptive oral sex: 0.35 (0.16–0.76)
Chen, 2023 [28]	Childhood sexual abuse: 4.25 (1.81–9.98)
Choi, 2004 [29]	UIAI: No Beijing residence card: 1.74 (1.13–2.68) 6 + sex-partners (vs. 1–5): 2.02 (1.30–3.15) STI ever: 2.87 (1.77–4.65) Never tested for HIV: 1.78 (1.03–3.06) URAI: 6 + sex-partners (vs. 1–5): 1.73 (1.12–3.35) STI ever: 1.78 (1.10–2.88) Never tested for HIV: 1.81 (1.02–3.19) # preventive services used prior 2 years: 1.84 (1.23–2.77)
Hentges, 2023 [34]	Age: 0.94 (0.89–0.99) Schooling (low < 12 years): 1.55 (0.99–2.40) No previous STI counseling: 1.51 (1.05–2.17) Non-condom use at sexual debut: 3.05 (2.12–4.40)
Lau, 2013 [40]	UAI with any man: Completed university: 0.22 (0.05–0.96) Regular partners only: 0.05 (0.01–0.48) Condoms not always available: 13.90 (4.94–39.10) Transactional sex: 2.90 (1.07–7.85) Agree that MSM in Shenzen would not always insist on condom use: 3.30 (1.24–8.78) Agree that MSM in Shenzen would certainly agree to use condom if the participant insist on doing so: 0.04 (0.01–0.29). UAI with casual or commercial partner: 6–10 partners in previous 6 mo. (vs. >6): 5.44 (1.62–18.25) > 10 partners (vs. >6): 9.21 (2.24–37.86) Condoms not always available: 5.30 (2.31–12.16) Agree that MSM in Shenzen would certainly agree to use condom if the participant insist on doing so: 0.24 (0.09–0.67) Search sex-partners at brothels: 5.12 (1.56–8.28)
Lau, 2014 [41]	Low education level (not data shown) Always drink alcohol before sex: 4.91 (1.53–15.75) Use of psychotropic substances: 3.23 (1.09–9.57) Can find someone to share sexual orientation: 4.00 (1.20-11.73) Disclosure sexual orientation to none or only some family members: 3.66 (1.41–9.53)
Maksut, 2016 [45]	Old men who have sex with men = Internalized homophobia: 0.75 (0.68, 0.83)
Morell-Mengual, 2021 [26]	Sexual assertiveness: 0.89 (0.84–0.94) Self-stem: 1.07 (1.01–1.13) Physical sensations attraction: 1.17 (1.08–1.26) Acquisition and negotiation: 0.72 (0.61–0.86) Impulse control: 0.76 (0.64–0.86) Fear of rejection: 0.82 (0.72–0.94)
Pham, 2015 [47]	Age of sex debut (17 + vs. <17) = aPR: 1.37 (1.04, 1.80) Selling sex = aPR: 1.88 (1.32, 2.49) Buying sex = aPR:1.55 (1.05, 2.27) Alcohol use (< 1x/week vs. ≥ 1/week) = aPR:1.60 (1.09–2.36)
Raymon, 2009 [50]	Age: 0.91 (0.8–1.0) HIV test prior 6 mo.: 2.81 (1.2–7.4) Gay: 9.92 (3.2–30.2) Heat of the moment: 5.72: (2.2–15.2).
Rocha, 2020 [51]	< 25 years: married: 2.61 (1.40–4.85) Practicing any religion: 1.97 (1.18–3.27) History of sexual violence: 2.03 (1.10–3.76) 5 + years younger partner (ref: 5 + years older): 2.01 (1.05–3.84) Same age partner (ref: 5 + years older): 2.40 (1.31–4.40) 6 + sex-partners (ref: ≤1): 2.63 (1.35–5.14) Stable partner: 2.04 (1.16–3.59) Sex only with men prior 6 mo.: 3.81 (2.04–7.11) Condom use at debut: 2.10 (1.25–3.56)
Sharma, 2018 [53]	Year of birth 1980–1989 (ref. 1928–1959): 2.8 (1.4–5.5) Functional knowledge of HIV prevention strategies: 1.3 (1.1, 1.4)
Stack, 2016 [54]	UAI at least once= College or higher: 0.68 (0.57–0.80) High income: 1.33 (1.04–1.70) Bisexual (ref. gay): 0.80 (0.65–0.98) Monogamous relationship: 1.68 (1.33–2.12) UAI > 5x/week: 1.70 (1.34–2.17) Frequent UAI= Age median: 0.98 (0.97–0.99) Bisexual (ref. gay): 0.50 (0.33–0.77) Monogamous relationship: 4.23 (3.05–5.86)
Vargas, 2018 [58]	UAI = aPR: 0.66 (0.49–0.87) History of syphilis = aPR: 1.59 (1.23–2.06) > High school = aPR: 1.38 (1.03–1.8) 2–4 sex-partners (ref. 0–1) = aPR: 1.73 (1.09–2.72)
Yi, 2015 [59]	Age 25+: 1.77 (1.09–2.86) Quality of life: good/very good: 4.37 (1.79–5.67) Illicit drug use: 5.76 (1.65–10.09) Use of lubricants: 2.85 (1.07–8.12)
	**Factors associated with perceived risk for HIV acquisition (different from sexual HIV exposure)**
Chard, 2017 [27]	Age: United States: 0.97 (0.95–0.99) 12 + years of education: Canada: 1.83 (1.06–3.16) Minority race: South Africa: 0.53 (0.3–0.91) Drug use prior 12 mo.: Canada 2.6 (1.67–4.05); South Africa 1.78 (1.22–2.58); UK: 2.23 (1.41–3.52); United States 1.85 (1.16–2.95) In a relationship (ref. single): Canada 0.54 (0.35–0.86); UK: 0.38 (0.24–0.6); United States 0.5 (0.31–0.82) Age at sex debut with man: Australia: 0.95 (0.9–0.99); Brazil: 0.94 (0.9-1.00) Percent of unprotected versus all anal intercourse partners in previous year: 0.5 (0.3–0.84) Years since most recent HIV test: Thailand 1.18 (1.05–1.31); UK: 0.92 (0.87–0.98)
Guillen-Diaz-Barriga, 2023 [31]	PrEP eligibility: 10.9 (2.5–47.9) Anticipated risk compensation: 3.0 (1.5–7.9) PrEP awareness: 4.9 (1.9–12.9); aOR: 35.9 (3.9–32.4) PEP awareness: 3.9 (1.5–9.2)
Hall, 2018 [33]	Transactional sex: 2.56 (1.01–6.45) Recent STI: 2.34 (1.32–4.12) Experienced more social isolation: 1.62 (1.01–2.60)
Luz, 2021 and Torres, 2019 [43], [56]	18–24 years: 0.72 (0.66–0.79) Non-white: 1.21 (1.12–1.31) Low income: 1.11 (1.02–1.21) Low education: 1.11 (1.02–1.21) Gay: 1.28 (1.12–1.47) Steady partner: 0.78 (0.71–0.85) Binge drinking: 1.15 (1.05–1.25) Stimulant use: 1.51 (1.38–1.66) STI: 2.40 (2.17–2.66)
MacKellar, 2007 [44]	Asian: 1.75 (1.19–2.59) Hispanic: 1.40 (1.10–1.78) Low education: 1.82 (1.47–2.25) HIV negative test prior year: 0.76 (0.63–0.92) 20 + lifetime male sex-partners: 1.60 (1.32–1.95) Injecting drugs: 1.73 (1.02–2.93) Previous STI: 1.77 (1.44–2.16) HIV-infected unaware: 2.70 (2.01–3.63)

aPR = Adjusted prevalence ratio; PEP: Post-exposure prophylaxis; PrEP = Pre-exposure prophylaxis; cis-MSM = cisgender men who have sex with men; STI: Sexually transmitted infections; UAI = Unprotected anal intercourse; UIAI = unprotected insertive anal intercourse; URAI = unprotected receptive anal intercourse

## Discussion

In the present systematic review, we aimed to identify the relationship between the perceived risk of acquiring HIV and sexual HIV exposure. We found evidence of an association between high perceived risk of HIV acquisition and sexual HIV exposure from most studies. Moderate or high perceived risk for HIV acquisition was associated with high sexual HIV exposure, and vice versa. Nevertheless, the definition of high sexual HIV exposure has changed over 40 years since the start of the HIV epidemic. For example, none of the studies did a differential analysis based on the estimated per-act probability of acquiring HIV from sexual exposure (i.e., receptive vs. insertive condomless anal sex) [67], PrEP use status, or the majority considered high sexual HIV exposure as a mixed result between substance use, condomless anal sex, number of sexual partners, etc. A recent study conducted in Brazil found that PrEP moderates the association between high perceived risk of HIV acquisition and sexual HIV exposure, resulting in no significant association between perceived risk of acquiring HIV and sexual HIV exposure among PrEP users [68]. More studies evaluating this association after WHO’s PrEP recommendation are needed [60], as sexual behavior and perceived risk for HIV acquisition among sexual and gender minorities could evolve and change due to the high efficacy of PrEP and treatment as prevention (TasP) in preventing sexual HIV transmission.

Though the studies reported on a wide range of participants, they had few representations of some populations, such as people aged 60 + years. We found no studies with transgender men participants, and only two included non-binary people. Tordoff et al. [69] found that transgender or non-binary people partnered with cisgender people could have worse health outcomes, such as higher self-reported HIV prevalence, history of STI, less HIV testing, or PrEP use than cisgender or transgender people. These findings indicate that more research is needed to understand perceived risk and sexual HIV exposure risk among sexual and gender minorities from low- and middle-income countries and among transgender, non-binary, and gender-diverse persons.

Perceived risk for HIV acquisition was assessed solely from the probability self-perspective. Risk perception is a complex construct, and an accurate assessment should consider various components [70]. These are deliberative component (i.e., evaluating the likelihood of acquiring HIV when having unprotected sex), affective component (i.e., experiencing fear or concern when considering potential health consequences), and experiential component (i.e., “gut-level reactions” due to previous experience with people living with HIV) [13, 71]. Perceived risk for HIV acquisition requires the use of validated and adapted instruments for each context and population (i.e., Perceived Risk for HIV Infection Scale [63], HIV Perceived Scale [64], etc.). Single-item or study-specific scales limit the ability to reflect variability in individual perceptions based on their deliberative, affective, or experiential components, so these assessments likely have insufficient validity to support their results. Therefore, perceived risk for HIV acquisition assessments should include a comprehensive evaluation to reach accuracy and then design better interventions to improve perception among the most vulnerable populations to HIV. Addressing the relationship between perceived risk for HIV acquisition and sexual HIV exposure assessments might include in-depth interviews and specific questions [72], as well as self-administration questionnaires to ensure confidentiality and anonymity [73]. Though we recognize that no single instrument will capture all dimensions of a construct or will apply to all populations, we encourage future research to evaluate currently available validated instruments and their applicability to one’s study. Moreover, for perceived risk, we encourage future studies to broaden risk assessment from its cognitive component to assess its affective, behavioral, and phychosocial components [71, 74].

We also found various definitions and assessments for sexual HIV exposure and perceived risk for HIV acquisition. Only a third of the studies used a validated instrument to assess sexual HIV exposure or perceived risk, and all other studies used one-question or study-specific scales without providing basic validation parameters. This diversity hampers consistency, comparability, replicability, content validity, and an accurate measurement of complex constructs such as sexual behavior or perceived HIV risk while also making the description of behaviors to be addressed in prevention or other interventions more complex [75]. For example, most studies collected sexual HIV exposure or perceived risk for HIV acquisition either with categorical or dichotomous variables or recategorized continuous variables into categories or dichotomous data for analysis. However, dichotomization or categorization could lead to loss of statistical power, reduced ability to detect nonlinear effects, introduce classification biases, and lack of consistency in selected cutoff points [76]. In this sense, studies should use cross-cultural and adapted instruments for their setting and population. This increases the validity and consistency of their findings, as well as the comparability and replicability with other populations. For example, the Perceived Risk of HIV Scale, created in the USA in English [64], was used in Brazil after a proper validation process [57]. Additionally, instruments to assess perceived risk for HIV acquisition should include the affective component of risk perception, which was notably absent in the studies included in this review [71]. One way to minimize recall and social desirability biases would be to include another measure as a point of comparison [77]. An additional measure of sexual exposure, for example, could be obtained with experience sampling or daily diary methods, both of which are designed to capture people’s experiences in real time and, therefore, less prone to recall bias [78]. Additionally, recall periods should be limited to the prior month, including mood assessment, for accurate data [79].

Self-evaluation of sexual behavior could be biased by recall and social desirability, mainly if an interviewer assesses it. Recall bias in retrospective studies is frequent due to participants’ ability to accurately remember and report past events [80], especially when obtaining information on health-related behaviors [81]. Also, the influence of mood at the time of any assessment can distort how people remember past events, so cognitive biases may cause them to reflect on events more positively or negatively than how they occurred. For example, the influence of prior expectations on the interpretation of past events and the difficulty in recalling specific events compared to more general events [82]. Additionally, the reviewed studies encompassed different timeframes for participants to identify, increasing the inaccuracy in recognizing behavior as more time passes [81]. Moreover, when studies assess sexual behavior, there are challenges associated with social desirability bias. These challenges become particularly important considering the social stigma attached to certain sexual practices, such as same-sex intercourse, anal sex, recreational drug use during sexual encounters, or having multiple sexual partners [72]. Additionally, social desirability bias could make individuals portray themselves positively to others, resulting in denial or underestimating behaviors or traits they perceive as socially undesirable or stigmatizing [73]. For example, high internalized homo or transphobia, resulting from long-term social rejection, are associated with mental health problems, low use of HIV preventive methods, or more frequent sexual HIV exposure [83, 84].

Finally, when it comes to social or behavioral research (e.g., sexual behavior or perceived risk for HIV acquisition), researchers should consider the influence of intersectionality on the decisions or behavior of specific populations [85]. Sexual and gender minorities, who are at increased vulnerability of acquiring HIV, build their identity and shape their perception and behavior under the influence of social and structural obstacles from very early ages as the result of their ethnicity, socio-economic status, stigma, discrimination, lack of access to education or work, among others [86, 87]. Therefore, the perceived risk for HIV acquisition or sexual HIV exposure among these minorities is not only a result of individual responsibility but also of those social identities that place them in subordination or vulnerability (e.g., being under socio-economic vulnerability, self-identified as trans women or Black/Latinx race/ethnicity) compared to other privileged social identities (e.g., being White, cisgender men, heterosexual, and highly educated) [88]. In this sense, 38.5% of the reviewed studies did not report participants’ ethnicity and how the perceived risk for HIV acquisition or sexual HIV exposure might be different according to their race or ethnicity. Assessment of perceived risk or sexual HIV exposure among sexual and gender minorities could be constructed under an intersectionality framework to achieve accurate outcomes. This reinforces the importance of the collection of race/ethnicity data on future studies.

Our review has limitations:1) most of the studies included only cis-MSM, predominantly white, and from high-income countries, which limits the generalizability to other sexual and gender minorities in low or middle-income countries; 2) most of the studies were cross-sectional, so they could not capture the temporal dynamics of the relationship and the causality between perceived risk for HIV acquisition and sexual HIV exposure; 3) as mentioned before, most of studies asked past sexual behavior which could be biased by recall and social desirability; 4) we did not collect information on intersectional aspects associated with perceived risk for HIV acquisition or sexual HIV exposure; and 5) we did not include qualitative research studies, which could have provided additional insights and understanding of the reasons and reasoning behind perceived risk and sexual HIV exposure. These limitations highlight the complexity of the relationship between perceived risk for HIV acquisition and sexual HIV exposure, suggesting that a combination of individual, social, and contextual factors can influence risk perception and sexual HIV exposure.

## Conclusions

We found evidence of an association between high perceived risk of HIV acquisition and sexual HIV exposure. Our results may be useful for the development of prevention and education strategies to address known risk behaviors and underlying factors affecting risk perception. Having an adequate perception of risk aligned with behavior is crucial for prevention, informed decision-making, access to education and resources, reduce stigma and discrimination (by gaining an accurate understanding of how the virus is transmitted and practicing safe sex, stereotypes, and misconceptions can be avoided), and promote self-care and personal well-being.

### Supplementary Information

Supplementary Material 1.

## Data Availability

No datasets were generated or analysed during the current study.

## Abbreviations

HIV: Human immunodeficiency virus
LGBTQIA+: Lesbian, gay, bisexual, transgender, queer, intersexual, asexual and more
Cis-MSM: Cisgender men who have sex with men
PrEP: Pre-exposure prophylaxis
TasP: Treatment as prevention
TW: Transgender women
WHO: World Health Organization

## Footnotes

1 We will use *sexual HIV exposure* for sexual HIV risk following recommendations from UNAIDS Terminology Guidance 2015 (https://www.unaids.org/sites/default/files/media_asset/2015_terminology_guidelines_en.pdf) and People First Charter: recommended terminology for research and publications related to HIV (https://img1.wsimg.com/blobby/go/307bf032-fd32-46de-894d-184dd697d7d1/People%20first%20charter%20language%20v3%2019042022.pdf).
