# Supplementary material for: Perceived risk for HIV acquisition and sexual HIV exposure among sexual and gender minorities: a systematic review
Source: BMC Infect Dis. 2024 Jun 10;24:574. doi: 10.1186/s12879-024-09456-0 (PMC11163805; doi:10.1186/s12879-024-09456-0)
Supplement: Supplementary file 1 — Supplementary Material 1. [file 12879_2024_9456_MOESM1_ESM.docx]

**ADDIONAL FILE 1. SEARCH KEYS**

*PubMed*

(("perceived risk" OR "perceived HIV risk"  OR "perceived HIV-risk" OR "HIV-perceived risk" OR "risk perception" OR "HIV-risk perception"  OR "perception of HIV risk"  OR "risk characterization" OR "perceptions of HIV threat" OR "self-perception of HIV risk" OR "HIV self-perception risk" OR "perceived likehood of getting HIV"  OR "perceived chances of getting HIV" OR "perceived likehood of acquiring HIV"  OR "perceived chances of acquiring HIV"  ) AND ("risk behavior" [Title/Abstract] OR "HIV-risk behavior" [Title/Abstract] OR  "current HIV risk" [Title/Abstract] OR  "current risk" [Title/Abstract] OR "actual risk" [Title/Abstract] OR "behavioral risk" [Title/Abstract]  OR "sexual risk" [Title/Abstract]  OR "sex risk" [Title/Abstract] OR "calculated risk" [Title/Abstract] OR "objective risk" [Title/Abstract] OR condomless [Title/Abstract] OR "condomless sex" [Title/Abstract] OR "condomless anal sex" [Title/Abstract] OR "condomless anal receptive sex" [Title/Abstract] OR "condomless intercourse" [Title/Abstract] OR "condomless anal intercourse" [Title/Abstract] OR "condomless anal receptive intercourse" [Title/Abstract] OR "vaginal sex" [Title/Abstract] OR "condomless vaginal sex" [Title/Abstract] OR "unprotected vaginal sex" [Title/Abstract] OR bareback [Title/Abstract] OR barebacking [Title/Abstract] OR "unprotected sex" [Title/Abstract] OR "unprotected anal sex" [Title/Abstract] OR "unprotected anal receptive sex" [Title/Abstract] OR "unprotected intercourse" [Title/Abstract] OR "unprotected anal intercourse" [Title/Abstract] OR "unprotected anal receptive intercourse" [Title/Abstract] OR "anal sex" [Title/Abstract] OR "anal receptive sex" [Title/Abstract] OR "anal intercourse" [Title/Abstract] OR "anal receptive intercourse" [Title/Abstract] OR "sex without a condom" [Title/Abstract]) AND (HIV [Title/Abstract] OR "human immunodeficiency virus" [Title/Abstract]))

*EMBASE*

('perceived risk':ti,ab,kw OR 'perceived hiv risk':ti,ab,kw OR 'perceived hiv-risk':ti,ab,kw OR 'hiv-perceived risk':ti,ab,kw OR 'risk perception':ti,ab,kw OR 'hiv-risk perception':ti,ab,kw OR 'perception of hiv risk':ti,ab,kw OR 'risk characterization':ti,ab,kw OR 'perceptions of hiv threat':ti,ab,kw OR 'self-perception of hiv risk':ti,ab,kw OR 'hiv self-perception risk':ti,ab,kw OR 'perceived likehood of getting hiv':ti,ab,kw OR 'perceived chances of getting hiv':ti,ab,kw OR 'perceived likehood of acquiring hiv':ti,ab,kw OR 'perceived chances of acquiring hiv':ti,ab,kw) AND ('risk behavior':ti,ab,kw OR 'hiv-risk behavior':ti,ab,kw OR 'current hiv risk':ti,ab,kw OR 'current risk':ti,ab,kw OR 'actual risk':ti,ab,kw OR 'behavioral risk':ti,ab,kw OR 'sexual risk':ti,ab,kw OR 'sex risk':ti,ab,kw OR 'calculated risk':ti,ab,kw OR 'objective risk':ti,ab,kw OR condomless:ti,ab,kw OR 'condomless sex':ti,ab,kw OR 'condomless anal sex':ti,ab,kw OR 'condomless anal receptive sex':ti,ab,kw OR 'condomless intercourse':ti,ab,kw OR 'condomless anal intercourse':ti,ab,kw OR 'condomless anal receptive intercourse':ti,ab,kw OR 'vaginal sex':ti,ab,kw OR 'condomless vaginal sex':ti,ab,kw OR 'unprotected vaginal sex':ti,ab,kw OR bareback:ti,ab,kw OR barebacking:ti,ab,kw OR 'unprotected sex':ti,ab,kw OR 'unprotected anal sex':ti,ab,kw OR 'unprotected anal receptive sex':ti,ab,kw OR 'unprotected intercourse':ti,ab,kw OR 'unprotected anal intercourse':ti,ab,kw OR 'unprotected anal receptive intercourse':ti,ab,kw OR 'anal sex':ti,ab,kw OR 'anal receptive sex':ti,ab,kw OR 'anal intercourse':ti,ab,kw OR 'anal receptive intercourse':ti,ab,kw OR 'sex without a condom':ti,ab,kw) AND (hiv:ti,ab,kw OR 'human immunodeficiency virus':ti,ab,kw)

*LILACS*

(("perceived risk" OR "perceived HIV risk"  OR "perceived HIV-risk" OR "HIV-perceived risk" OR "risk perception" OR "HIV-risk perception"  OR "perception of HIV risk"  OR "risk characterization" OR "perceptions of HIV threat" OR "self-perception of HIV risk" OR "HIV self-perception risk" OR "perceived likehood of getting HIV"  OR "perceived chances of getting HIV" OR "perceived likehood of acquiring HIV"  OR "perceived chances of acquiring HIV") AND ("risk behavior" OR "HIV-risk behavior" OR  "current HIV risk" OR  "current risk" OR "actual risk" OR "behavioral risk" OR "sexual risk" OR "sex risk" OR "calculated risk" OR "objective risk" OR condomless OR "condomless sex" OR "condomless anal sex" OR "condomless anal receptive sex" OR "condomless intercourse" OR "condomless anal intercourse" OR "condomless anal receptive intercourse" OR "vaginal sex" OR "condomless vaginal sex" OR "unprotected vaginal sex" OR bareback OR barebacking OR "unprotected sex" OR "unprotected anal sex" OR "unprotected anal receptive sex" OR "unprotected intercourse" OR "unprotected anal intercourse" OR "unprotected anal receptive intercourse" OR "anal sex" OR "anal receptive sex" OR "anal intercourse" OR "anal receptive intercourse" OR "sex without a condom") AND (HIV OR "human immunodeficiency virus"))
